# Full-Length Genome Sequences of Two Chinese Porcine Circovirus Type 3 Strains, NWHEB21 and NWHUN2

**DOI:** 10.1128/genomeA.00062-18

**Published:** 2018-02-15

**Authors:** Can Liu, Shasha Chen, Fanwei Meng, Rui Chen, Zhigang Zhang, Enqi Du, Qinghong Xue

**Affiliations:** a Shaanxi Innolever Biotechnology Co. Ltd., Yangling, Shaanxi, China; b Yangling Research Center for Immunobiological Products, Yangling, Shaanxi, China; c China Institute of Veterinary Drug Control, Beijing, China

## Abstract

Two porcine circovirus 3 (PCV3) strains, named NWHEB21 and NWHUN2, were identified in heart and brain tissues of aborted piglets. Their complete genome sequences were sequenced and analyzed to further characterize PCV3 in China and worldwide.

## GENOME ANNOUNCEMENT

Porcine circovirus 3 (PCV3), like nonpathogenic PCV1 and pathogenic PCV2, belongs to the genus *Circovirus* (1). For the first time, PCV3 was reported to be found on a farm with porcine dermatitis and nephropathy syndrome (PDNS) in North Carolina, USA, in 2015, with a 10.2% increase in sow deaths and 1.19 more mummies per litter. It was confirmed that PCV2, porcine reproductive and respiratory syndrome virus (PRRSV), and swine influenza virus (SIV) tests were negative, based on immunohistochemistry (IHC) and fluorescent quantitative PCR (qPCR), while heart, lung, and lymph node samples tested positive for PCV3 by qPCR and immunofluorescence assay (IFA); moreover, no other viral genetic sequences were found in metagenomic sequences (2).

Reports from 2016 to 2017 showed that PCV3 strains were prevalent globally, including in the United States, China, South Korea, Poland, Brazil, and the United Kingdom, accompanied by asymptomatic or reproductive disorders, respiratory disorders, congenital tremor, and diarrhea (3–9), among which the retrospective detection results from the United Kingdom showed that some samples collected in 2009, 2004, and even 2002 tested positive for PCV3 (10). Thereupon, we collected aborted piglets from several farms in the Hebei and Hunan Provinces; subsequently, PCV3 viral nucleic acids were found, with a viral load of up to 10^6.5^ copies/μl in both myocardium and brain tissues. Whole-genome sequencing was performed on the positive tissues to obtain two PCV3 whole-genome sequences of strains named NWHEB21 and NWHUN2.

The complete genome sequences of PCV3 NWHEB21 and NWHUN2 were 2.0 kb each, and the nucleotide sequence homology between the two strains was 99.1%; the nucleotide sequences were 97.5% and 97.6% homologous with that of the GD-2016 strain in GenBank, respectively. Another 32 strains of PCV3 from around the world had homologies of 99.0 to 99.3% with NWHEB21 and NWHUN2. Based on the phylogenetic tree of PCV3 NWHEB21, NWHUN2, and the other 33 strains of PCV3, PCV3 GD-2016 was found as a single branch, and the remaining 34 strains of PCV3 can be further divided into 5 branches (data not shown). The Rep and Cap genes of PCV3 NWHEB21 and NWHUN2 consist of 891 and 645 nucleotides, respectively. Compared with the first published PCV3 strain, 29160, there were 19 nucleotide variations in NWHEB21 and 18 variations in NWHUN2, of which 10 nucleotides were the same variation in the two of them; further, 8 nucleotides were identical within the coding frame of Cap and corresponded to 4 amino acid mutations (A24V, R27K, T77S, and L150I).

The sequence analysis of PCV3 NWHEB21 and NWHUN2, as well as a comparison with other sequences in GenBank, could facilitate an understanding of the characteristics and pattern of PCV3 genetic variation and provide theoretical support for the prevention and control of this infectious disease.

### Accession number(s).

The genome sequences of the porcine circovirus type 3 strain NWHEB21 and strain NWHUN2 have been deposited to GenBank under the accession no. MG564174 and MG564175, respectively.
